# 

**Published:** 1882-02

**Authors:** 


### Pamphlets.

Obstetric and Gynæcological Literature, 1876-1880. Addresses on Obstetrics and Diseases of Women, American Medical Association, 1881. James R. Chadwick, Boston.

Annual Report of the Surgeon-General of the United States Army, 1881.

Dedication of the Hall of the Boston Library Association. Address by the President, Dr. O. W. Holmes.

Third Annual Report of the Board of Health of the City of Atlanta, for the year 1881. W. S. Armstrong, M.D., President; Jas. B. Baird, M.D., Secretary.

On the Poisonous Properties of Quinine. By William O. Baldwin, M.D., of Montgomery, Ala. With remarks by J. Marion Sims, M.D. Reprint from Medical Gazette, Oct., 1881.

*The American Bulletin*, issued occasionally by the New

York Committee of the International Federation to Promote the Abolition of State Regulated Vice.

Anæsthetics Medico-Legally Considered. By J. G Johnson, M.D., Brooklyn, N. Y. Reprint from the Bulletin of the Medico-Legal Society of New York.

The Prevention of Syphilis. An address prepared at the request of the Philadelphia County Medical Society, and read before it December 14, 1881. By J. William White, M.D. Reprint from the Philadelphia Medical Times.

